# Sarcoidosis limbic encephalitis: A case report

**Published:** 2017-07-06

**Authors:** Moussa Toudou-Daouda, Hamid Assadeck, Boubacar Efared

**Affiliations:** 1 Department of Neurology, Hassan II University Hospital, Fez, Morocco; 2 Department of Medicine and Medical Specialties, Niamey National Hospital, Niamey, Niger; 3 Department of Pathology, Hassan II University Hospital, Fez, Morocco

**Keywords:** Sarcoidosis, Magnetic Resonance Imaging, Limbic Encephalitis

Sarcoidosis is a rare multisystem chronic granulomatous, with unknown etiology. Neurological involvement in sarcoidosis is rare and occurs in 5 to 15% of cases.^1^ Limbic structures involvement in sarcoidosis is unusual and uncommon. Herein, we report a case of limbic encephalitis (LE) as the first manifestation of neurosarcoidosis.

A 39-years-old man with no known past medical history was admitted to our department for short-term memory disorders associated with behavioral type of irritability and agitation behavior occurred 1 month before consultation. 

On admission, he was a patient with anxiety and logorrhea, marked disorientation and cognitive impairment with deficit in free recall, difficulty in learning, and calculation deficit with a Folstein Mini-Mental State Examination (MMSE) score of 12/30 which is pathological. In addition, the examination showed an inflammatory flattening of the root of the nose and gynecomastia. 

Nasofibroscopie showed inflammatory granulations causing stenosis of nasal cavities and deformed nasal septum. Cerebral magnetic resonance imaging (MRI) showed hyperintensities in right temporoinsular region on FLAIR and T2-weighted images (Figure 1, A and B) and on gadolinium enhanced T1-weigthed images, an enhancement and nodular leptomeningeal thickening in the basilar perimesencephalic cistern extended to the right temporal lobe, hypothalamus, and third ventricle (Figure 1, C and D). Electroencephalogram (EEG) revealed slowing of the basic rhythm with a frontotemporal intermittent rhythmic delta activity predominant in the right. Thoracic computed tomography (CT) scan showed mediastinal and hilar lymphadenopathies without parenchymal lung lesions. 

In systemic immunological tests antinuclear antibodies [anti-double stranded DNA (anti-dsDNA), anti-Sjögren’s-syndrome-related antigen A (anti-SSA), anti-SSB, and perinuclear anti-neutrophil cytoplasmic (p-ANCA) antibodies] were negative. Histological examination of nasal biopsy showed a granulomatous inflammation made of confluent granulomas with multinucleated giant and epithelioid cells surrounded by a rim of lymphocytes without caseous necrosis favoring the diagnosis of sarcoidosis. 

Study of cerebrospinal fluid (CSF) revealed meningitis with 19 white blood cells, of which 75% were lymphocytes, protein level of 0.37 g/l, chloride level of 125 mmol/l, and glucose level of 0.51 g/l. CSF cultures were negative. 

**Figure 1:**
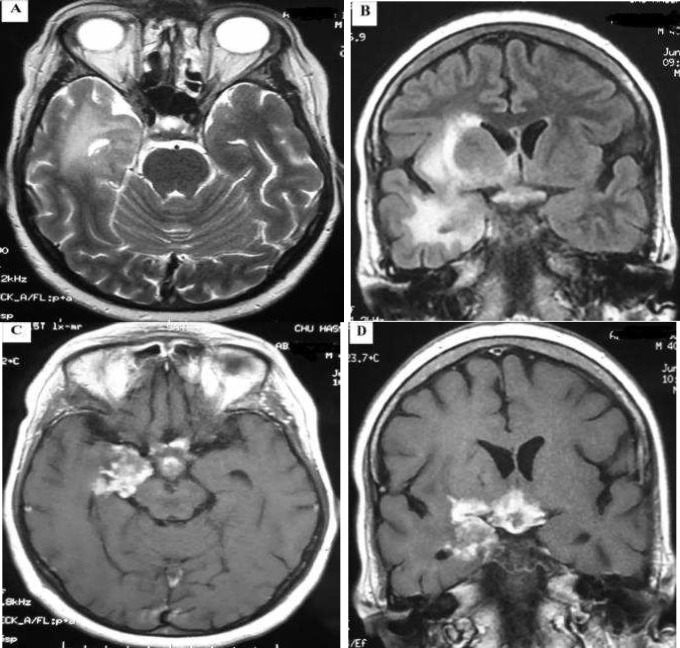
Cerebral magnetic resonance imaging (MRI) showing hyperintensities in the right temporoinsular region associated with an enhancement and nodular leptomeningeal thickening of the basilar peri-mesencephalic citern extended to the right temporal lobe, hypothalamus and walls of the third ventricle. (A) T2-weighted images, (B) FLAIR sequences, (C and D) gadolinium enhanced T1-weigthed images

The dosage of angiotensin converting enzyme in blood was high at 98 IU/l. The hypophysiogramme revealed gonadotropic failure, adrenocorticotropic failure, and thyroid failure with a slight hyperprolactinemia at 76.33 ng/ml. 

Serologies of syphilis, human immunodeficiency virus (HIV), and hepatitis B and C were negative as well as herpes simplex virus via polymerase chain reaction (HSV-PCR) in CSF. Assessment of mycobacterium tuberculosis in the gastric lavage fluid and intradermoreaction to tuberculin were normal.

At the end of analysis, a diagnosis of sarcoidosis LE was considered. The patient was treated with pulse intravenous methylprednisolone (1 g/kg/day for 5 days) followed by oral corticotherapy, associated with levothyroxine. The patient’s cognitive status improved with a Folstein MMSE score of 23/30 indicating a good clinical response to the oral corticotherapy; although the control EEG was not performed at this time.

LE is histologically defined as an inflammation-degeneration of limbic structures.^2^ Clinically, LE is characterized by an acute or subacute onset of short-term memory disorders, psychiatric disorders, confusional state, and temporal lobe or generalized epilepsy.^2^^,^^3^ Cerebral MRI plays an important role in the diagnosis of LE when it is positive by highlighting hyperintensities in the limbic regions on T2 and FLAIR sequences, such as the internal part of the temporal lobe, hippocampus and amygdala, cingulate gyrus, fornix, and hypothalamus.^3^

Many etiologies have been described in the literature with a predominance of infectious diseases and autoimmune encephalitis or paraneoplasms.^3^ Sarcoidosis is one of the uncommon etiologies of LE, and it is few described. The present case described a case of LE as the first manifestation of neurosarcoidosis which is a rare clinical entity. The clinical presentation of our patient was marked by neuropsychiatric and neuropsychological disorders that are the usual manifestations of LE. Although the signal abnormalities of cerebral MRI of the present case were unilateral, presented clinical signs suggested that the cerebral lesions were bilateral but asymmetrical. Functional cerebral imaging [single-photon emission computed tomography (SPECT) and fluorodeoxyglucose-positron emission tomography (FDG-PET)], the most sensitive radiological examination in the diagnosis of LE, should have been performed in our patient which could help to better visualisation of probable asymmetric lesions in the left limbic regions that have not been visualized by the cerebral MRI.^4^

Corticosteroid therapy constitutes the first line treatment of neurosarcoidosis,^5^ as the case of our patient. However, in cases of ineffectiveness, intolerance, or contraindications for corticosteroid therapy, immunosuppressive therapy and rituximab may be used as an alternative treatment.^5^

In conclusion, our observation shows the importance to search systematically neurosarcoidosis in the patients with hyperintensities in cerebral MRI on T2 and FLAIR sequences in the limbic regions associated with an enhancement and nodular leptomeningeal thickening.

## Notes:

**How to cite this article:** Toudou-Daouda M, Assadeck H, Efared B. Sarcoidosis limbic encephalitis: A case report. Iran J Neurol 2017; 16(3): 156-8.
